# The Soil Biota Composition along a Progressive Succession of Secondary Vegetation in a Karst Area

**DOI:** 10.1371/journal.pone.0112436

**Published:** 2014-11-07

**Authors:** Jie Zhao, Shengping Li, Xunyang He, Lu Liu, Kelin Wang

**Affiliations:** 1 Key Laboratory of Agro-ecological Processes in Subtropical Region, Institute of Subtropical Agriculture, Chinese Academy of Sciences, Changsha, Hunan, China; 2 Huanjiang Observation and Research Station for Karst Ecosystems, Chinese Academy of Sciences, Huanjiang, Guangxi, China; 3 Graduate University of Chinese Academy of Sciences, Beijing, China; Tennessee State University, United States of America

## Abstract

Karst ecosystems are fragile and are in many regions degraded by anthropogenic activities. Current management of degraded karst areas focuses on aboveground vegetation succession or recovery and aims at establishing a forest ecosystem. Whether progressive succession of vegetation in karst areas is accompanied by establishment of soil biota is poorly understood. In the present study, soil microbial and nematode communities, as well as soil physico-chemical properties were studied along a progressive succession of secondary vegetation (from grassland to shrubland to forest) in a karst area in southwest China. Microbial biomass, nematode density, ratio of fungal to bacterial biomass, nematode structure index, and nematode enrichment index decreased with the secondary succession in the plant community. Overall, the results indicated a pattern of declines in soil biota abundance and food web complexity that was associated with a decrease in soil pH and a decrease in soil organic carbon content with the progressive secondary succession of the plant community. Our findings suggest that soil biota amendment is necessary during karst ecosystem restoration and establishment and management of grasslands may be feasible in karst areas.

## Introduction

Karst landscapes account for about 12% of the Earth's land surface [1]. Ecosystems in karst areas are fragile and suffering extreme degradation in many regions of the world, such as southwest China, Southeast Asia (e.g., Vietnam and Thailand), and Central America (e.g., Mexico, Puerto Rico, Dominican Republic, and Cuba) [2]–[7]. Karst areas in southern and central Europe (e.g., Austria, Bosnia, Herzegovina, Croatia, Germany, Italy, Russia, and Spain [not all land cover of these countries are karst but there are karst areas in them]) also are facing significant land degradations [3]. One important example of this degradation is “karst rocky desertification”, which is characterized in part by shallow soils and exposed bedrock [1]. Karst rocky desertification results largely from anthropogenic activities such as irrational cultivation and extensive logging. Since the 1980s, karst rocky desertification has been recognized as a critical factor limiting the social and economic development of the karst region in southwest China [8]–[9].

The Chinese central and local governments have promoted the ecological restoration of karst areas in southwest China, and as a consequence many farmlands and plantations have been abandoned and allowed to recover naturally. Several studies have documented the succession of plant communities in the karst region in southwest China. For example, Zeng et al. (2007) identified the following six plant communities occurring during succession following the natural revegetation of areas suffering from rocky desertification: sparse grass, grass, shrub, liana-shrub, deciduous broad-leaved forest, and mixed evergreen and deciduous broad-leaved forest [8]. An earlier study had also identified six vegetation types: grass, grass-shrub, shrub, shrub-tree, tree forest, and climax mixed evergreen and deciduous broad-leaved forest [10]. Although the classification of the plant communities is somewhat different in these two studies, the succession from grass to shrub to forest is considered typical in this area [11]–[12].

The restoration of degraded karst areas in southwest China is currently evaluated based on the degree of forest vegetation establishment but the restoration of ecosystem services will also depend on the recovery of other organisms including soil organisms. Whether a secondary vegetation succession can promote the increment of biomass and abundance of soil biota in degraded karst areas is largely unknown, and this lack of knowledge limits our ability to understand and predict the functioning of such ecosystems.

In studying the succession of plant communities and soil biota, researchers frequently use space-for-time substitution, i.e., chronosequences [13]. In addition, researchers recognize two general types of succession, which are primary and secondary. Secondary succession is widespread and occurs at a high rate relative to primary succession [14]. In the past two decades, a number of studies have explored the relationship between secondary vegetation succession and soil biota. For example, vegetation succession can influence the composition of soil biota through resource and habitat changes [15]–[17], and the soil biota can affect the vegetation succession by selective feeding and by altering nutrient availability [18]–[19]. Few studies, however, have explored the changes in soil biota (especially nematodes) during secondary succession from grassland to other ecosystem types, and these studies have yielded inconsistent results concerning the relationship between vegetation succession and soil biota succession. For example, soil microbial biomass increased during the secondary succession of abandoned fields ranging in age from 1 to 60 years in east-central Minnesota [17] but declined across a secondary vegetation succession from short grassland to tall grassland to shrub-land in northwest France [20]. In addition, soil microbial biomass did not differ between early and late stages in a secondary succession from shrubland to forest in New Zealand [16].

In this study, we determined whether the progressive succession of the plant community is accompanied by the establishments of soil microbial and nematode communities in karst shallow soils; by progressive succession, we mean that the community becomes more complex, with increased species richness, community biomass, and food web trophic links. We explored the compositions of soil microbial and nematode communities along a secondary vegetation succession from grassland to shrubland to forest in the karst region in southwest China, where ecosystems have been severely disrupted and where the soils are very shallow. We test the hypothesis that progressive succession of the plant community should improve the soil habitat (because of increases in soil organic matter and nutrients) and thereby support the establishments of the soil microbial and nematode communities.

## Materials and Methods

No specific permits were required for the described field studies. No specific permissions were required for these locations/activities. The location is not privately owned or protected in any way and the field studies did not involve endangered or protected species.

### Site description

This study was carried out at the Huanjiang Observation and Research Station for Karst Ecosystems (107°51′–108°43′E, 24°44′–25°33′N), Chinese Academy of Sciences (CAS), Guangxi Province, China. The climate is subtropical monsoon with a distinct wet (from April to September) and dry season (from October to March). The mean annual temperature and precipitation are 18.5°C and 1,389 mm, respectively. The soil developed from a dolostone base and is calcareous.

Three plant communities (or ecosystems) were identified on the hillsides around the Huanjiang Observation and Research Station: grassland, shrubland, and forest. The main criteria used in selecting and designating these vegetation types were as follows: the plant community composition should be different, the disturbance history of the site should be known (based on information obtained from local farmers), and the environmental conditions (soil origin, altitude, slope orientation, and slope gradient) should be as similar as possible. All three vegetation types were situated in the middle of south-facing slopes with gradients ranging from 30 to 50°. The altitude ranged from 280 to 464 m. The grasslands were developed from managed forests which had been abandoned for 9 to 10 years, and wildfire had occurred three times since all of the trees had been harvested and replaced by grasses. The dominant plant in the grassland was *Miscanthus floridulu*. The shrublands were developed from managed forests which had been abandoned for 25 to 30 years, at which time all of the trees were removed (i.e., all of the wood was harvested). The common shrub species were *Loropetalum chinensis*, *Litsea coreana* lvl. var. *Sinensis*, and *Pittosporum tonkinense Gagnep*. The forest sites had previously been managed forests and had been abandoned 50 to 55 earlier. The dominant overstory species in the forest was *Cyclobalanopsis glauca*; the common understory species included *Litsea coreana* levl. var. *Sinensis* and *Loropetalum chinensis*.

In 2011, the three identified plant communities at nine sites were selected, among which each plant community including three sites (replicates). A 10×10 m plot was established at each of the grassland and shrubland site, while a 20×30 m plot was established at each of the forest site. Because of anthropogenic disturbances in this area over the past decades, no primary forest ecosystem on calcareous soil that developed from a dolomite base existed near the research station. Therefore, no primary forest ecosystems were included in the present study.

### Soil sampling and analysis

Soil was sampled in September 2012. Soil cores (2.5 cm in diameter, 5 cm in length) were taken at 0–5 cm and 5–10 cm depths from 15 random locations within each of the nine plots. The 15 cores of the same depth from each plot were combined to form one composite sample, giving three replicate samples for each combination of vegetation type and depth. The surface litter was carefully removed before soil cores were collected. Samples were brought to the laboratory in insulated boxes. Then, the soil was sieved to pass through a 2 mm sieve to remove the roots. The soil water content, microbial and nematode communities were determined on a fresh subsample. Another subsample was thereafter air-dried and used for determination of soil pH, soil organic C and total nitrogen.

Soil pH was determined in 1∶2.5 (w/v) soil: water suspensions, and soil water content (SWC %, g of water per 100 g dry soil) was measured by oven-drying the soil for 48 h at 105°C. Soil organic C (SOC, g kg^−1^ dry soil) was determined by dichromate oxidation, and total N (TN, g kg^−1^ dry soil) was measured with an ultraviolet spectrophotometer after Kjeldahl digestion [21].

Phospholipid fatty acids (PLFA) were extracted from 8 g fresh soil and were analyzed according to Bossio and Scow (1998) [22]. Concentrations of each PLFA were calculated relative to 19∶0 internal standard concentrations. Bacterial biomass was considered to be represented by 10 PLFAs (i15∶0, a15∶0, 15∶0, i16∶0, 16∶1ω7, i17∶0, a17∶0, 17∶0, cy17∶0, and cy19∶0), fungal biomass was considered to be represented by the PLFA 18∶2ω6,9, and actinomycete biomass was considered to be represented by three PLFAs (10 Me 16∶0, 10 Me 17∶0, and 10 Me 18∶0) [23]–[25]. Microbial biomass was considered to be represented by the 10 bacterial PLFAs and the one fungal PLFA. Other PLFAs such as 16∶1ω5, 18∶1ω7, and 18∶1ω9 were also used to analyze the soil microbial community.

Nematodes were extracted from 50 g of fresh soil using the Baermann funnel method [26]. After fixation in a 4% formalin solution, nematodes were counted with a differential interference contrast (DIC) microscope (ECLIPSE 80i, Nikon), and the first 100 individuals encountered were identified to genus. All nematodes were identified to genus when the sample contained fewer than 100 individuals. Nematodes were assigned to main trophic groups (bacterivores, fungivores, herbivores, omnivores, and predators) [27] and colonizer-persister guilds [28].

The microbial data were used to calculate a ratio of fungal biomass to bacterial biomass (F:B) and a cy/pre ratio of PLFAs [(cy17∶0+cy19∶0)/(16∶1ω7c+18∶1ω7c)]. The nematode data were used to calculate an enrichment index (EI), a structure index (SI), a maturity index (MI), and a plant-parasitic index (PPI) for each sample [29]–[30].

### Data analysis

Two-way ANOVAs were used to examine the main and interaction effects of plant community and soil depth on dependent variables describing soil physico-chemical properties and soil microbial and nematode communities. Soil physico-chemical properties and microbial community composition were also analyzed by transforming the data to their principal components (PCA) and analyzing these using ANOVAs [31]–[32]. Data were transformed (natural log, square root, or rank) to meet assumptions of normality and homogeneity of variance. Statistical significance was determined at *p*<0.05. ANOVAs and PCAs were performed using SPSS software (SPSS Inc., Chicago, IL). LSD was used to test differences among treatment means. Tamhane's T2 was used to test differences among treatments when variances of transformed data were unequal. Redundancy analysis (RDA) was used to determine the relationship between soil physico-chemical properties and microbial and nematode community indices, microbial biomass, or nematode abundance. Soil layer was converted to a nominal variable (0, 1) and was treated as a covariable [33]–[35]. Monte Carlo permutation tests were used to compute statistical significance. RDA was performed with CANOCO 4.5 software [33].

## Results

### Soil physico-chemical properities

Soil pH was highest in the grassland, lowest in the forest, and intermediate in the shrubland (Table 1). Soil organic carbon (SOC) was greater in the grassland than in the shrubland and forest. Soil C/N was greater in the grassland and shrubland than in the forest. Soil water content (SWC) and total nitrogen (TN) content, however, did not differ among the plant communities (Table 1).

**Table 1:** Soil physico-chemical properties at two depths in a grassland, shrubland, and forest in the degraded karst region of southwest China.

Property	Depth (cm)	Vegetation type					
		Grassland		Shrubland		Forest	
SWC (%)	0–5	47.3 (1.4)		54.9 (4.4)		44.7 (8.3)	
	5–10	43.2 (2.9)		45.8 (2.6)		40.1 (6.3)	
pH_WATER_	0–5	8.17 (0.02)	a^a^	7.53 (0.32)	b	6.89 (0.42)	c
	5–10	8.20 (0.03)		7.60 (0.31)		6.85 (0.33)	
TN (g/kg)	0–5	4.79 (0.22)		4.29 (0.20)		5.25 (0.79)	
	5–10	4.33 (0.07)		3.56 (0.14)		4.29 (0.73)	
SOC (g/kg)	0–5	96.9 (5.0)	a	67.8 (9.6)	b	56.5 (8.8)	b
	5–10	87.9 (6.1)		50.6 (13.6)		39.4 (6.9)	
C:N	0–5	20.4 (1.9)	a	16.1 (3.1)	a	10.8 (0.5)	b
	5–10	20.4 (1.7)		14.5 (4.3)		9.2 (0.5)	

a Means for a property (averaged over both soil depths) followed by different letters are significantly different (*p*<0.05) according to the LSD test.

SWC (%), pH, TN (g kg^−1^ dry soil), SOC (g kg^−1^ dry soil), and C:N stand for soil water content, soil pH, soil total nitrogen, soil organic carbon, and ratio of soil organic carbon to total nitrogen, respectively.

### Soil microbial community

**Figure 1:**
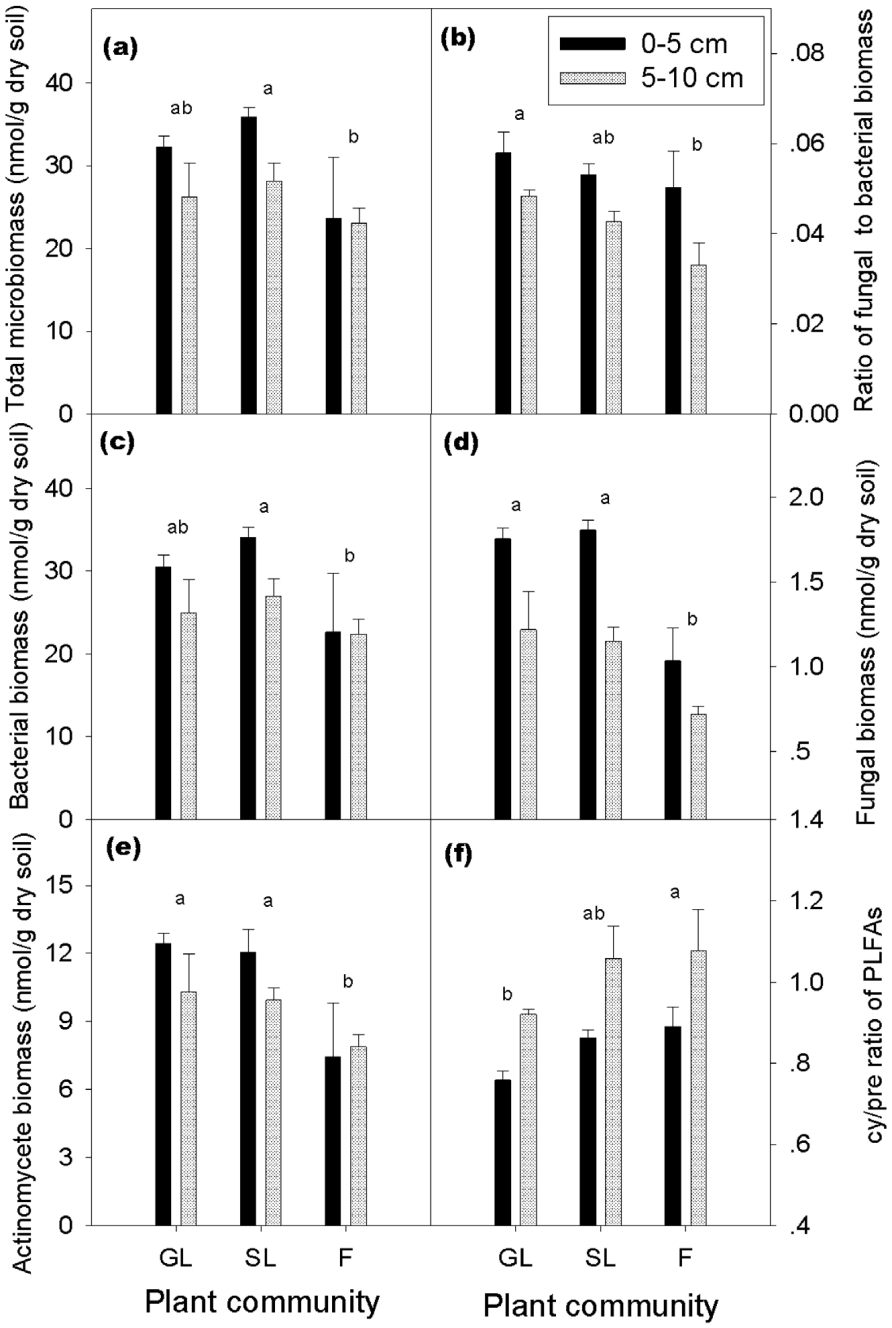
Characteristics of microbial communities (as determined by PLFA analysis) in the grassland (GL), shrubland (SL), and forest (F) at 0–5 and 5–10 cm soil depths. (a) Microbial biomass; (b) Ratio of fungal biomass to bacterial biomass; (c) Bacterial biomass; (d) Fungal biomass; (e) Actinomycetes biomass; and (f) cy/pre ratio. Bars indicate standard errors of means. Within each vegetation type, values with the same letters are not significantly different (*p>*0.05) according to the LSD test.

**Figure 2:**
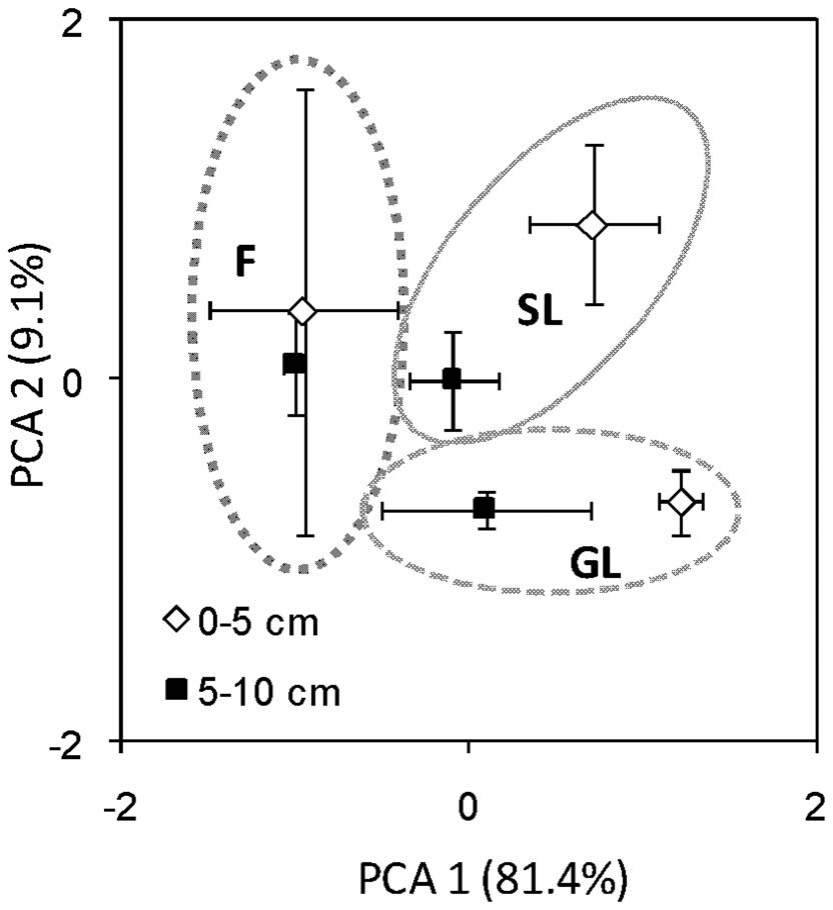
Principal component analysis (PCA) of microbial PLFA biomarkers of the grassland (GL), shrubland (SL), and forest (F) at 0–5 and 5–10 cm soil depth.

Microbial biomass and bacterial biomass were higher in the shrubland than in the forest (Fig. 1a and c). Fungal biomass and actinomycete biomass were higher in the grassland and shrubland than in the forest (Fig. 1d and e). The ratio of fungal to bacterial biomass (F:B) decreased gradually from grassland to shrubland to forest and was significantly higher in the grassland than in the forest (Fig. 1b). The cy/pre ratio of PLFAs increased from grassland to shrubland to forest (Fig. 1f) and was significantly higher in the forest than in the grassland (*p*<0.05) and tended to be higher in the shrubland than in the grassland (*p* = 0.058). In addition, ANOVA results of the transformed principal components of the soil microbial community showed that the microbial community structure of the forest differed from that of the grassland and shrubland (*p*<0.01) (Fig. 2). Moreover, the soil microbial community structure tended to differ between the grassland and shrubland (*p* = 0.069) (Fig. 2).

### Soil nematode community

**Figure 3:**
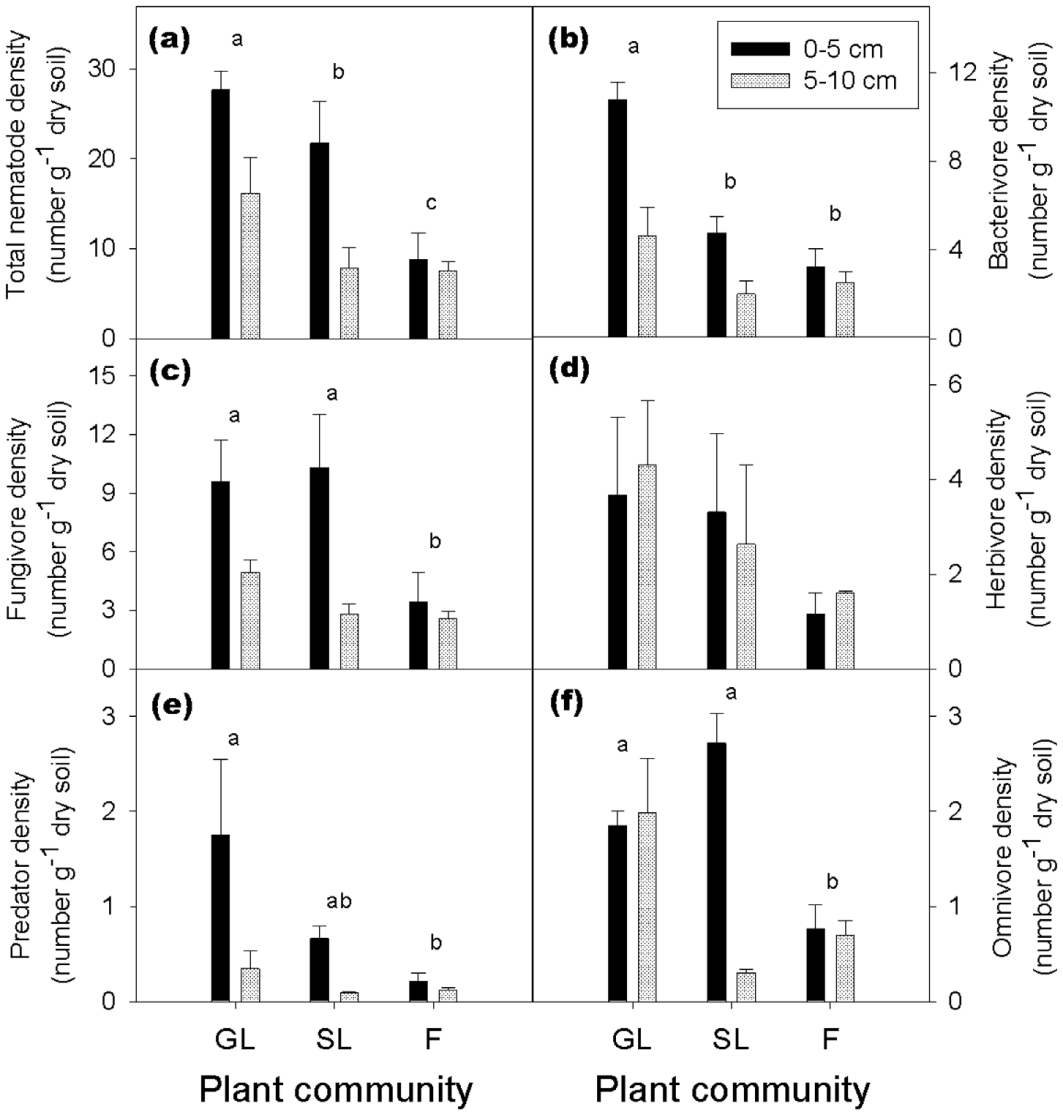
Densities of total nematodes and different nematode trophic groups in the grassland (GL), shrubland (SL), and forest (F) at 0–5 and 5–10 cm soil depth. (a) Total nematodes; (b) Bacterivores; (c) Fungivores; (d) Herbivores; (e) Predators; and (f) Omnivores. Bars indicate standard errors of means. Within each panel, values (averaged across both depths for each plant community) with the same or no letters are not significantly different (*p>*0.05) according to the LSD test.

Fungivores and bacterivores were the most abundant trophic groups (Fig. 3b and c). Total nematode density declined gradually from grassland to shrubland to forest at both 0–5 and 5–10 cm soil depths (Fig. 3a). Bacterivore density was greater in the grassland than in the shrubland and forest (Fig. 3b). Fungivore density and omnivore density were greater in the grassland and shrubland than in the forest (Fig. 3c and f); omnivore density in the shrubland, however, was very low at 5–10 cm depth (Fig. 3f). Predator density declined gradually from grassland to shrubland and forest and was significantly greater in the grassland than in the forest (*p* = 0.023) (Fig. 3e). Herbivore density declined gradually from grassland to shrubland to forest but did not significantly differ among the three ecosystems (Fig. 3d).

**Figure 4:**
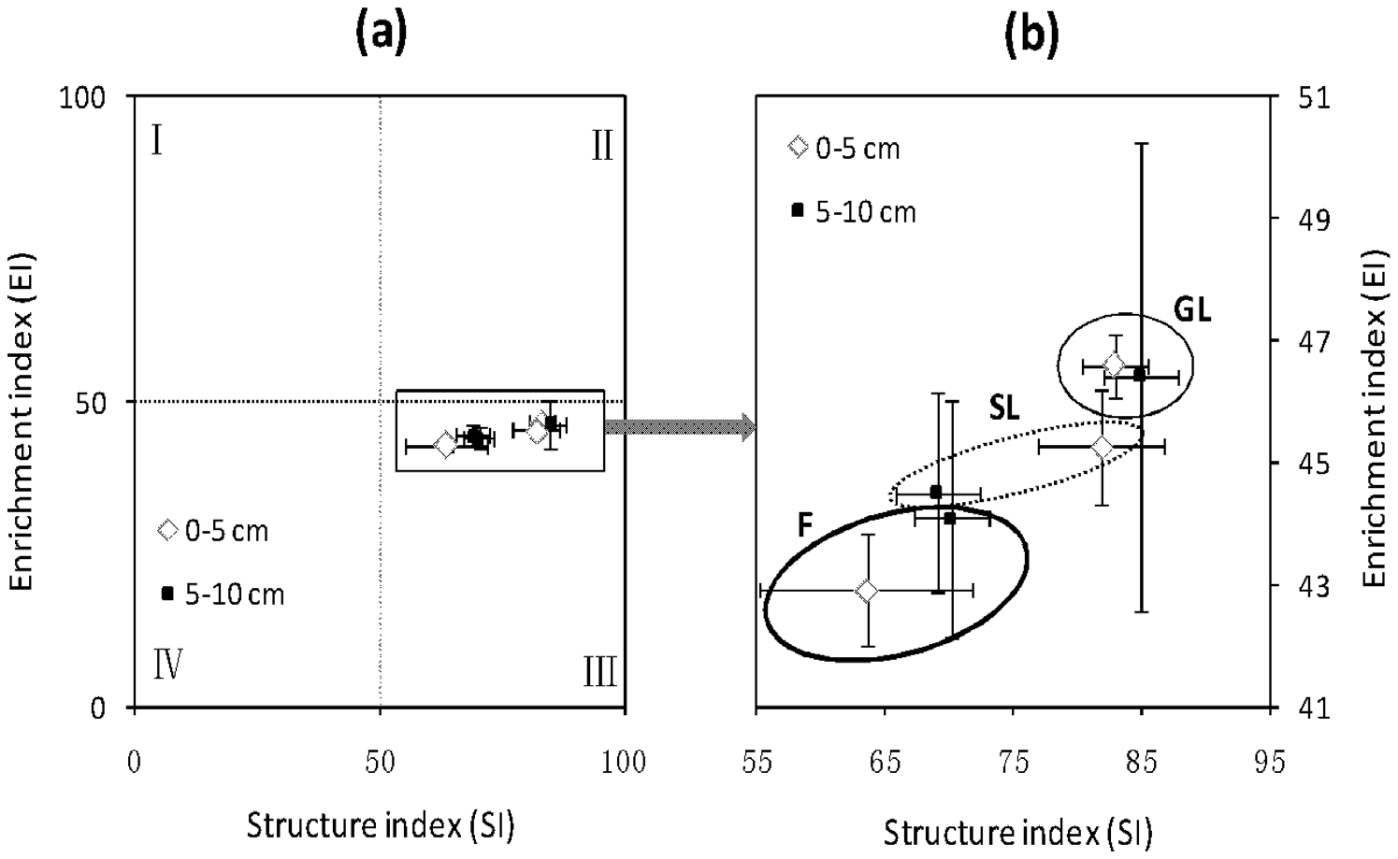
Weighted soil nematode faunal analysis at 0–5 depth and 5–10 cm depth in three vegetation types (GL, grassland; SL, shrubland; and F, forest). (a) Bi-plot with four quadrats; (b) Magnified quadrate-area of bi-plot A. Bars indicate the standard errors of means.

The weighted soil nematode fauna analysis showed that the nematode communities of all three ecosystems at both 0–5 and 5–10 cm soil depths projected onto quadrat III of the nematode fauna plot (Fig. 4a). The composition of the soil nematode community differed among the three ecosystems, and the value of the nematode structure index (SI) was greater in the grassland than in the forest (*p* = 0.003) (Fig. 4b). The SI value of the shrubland was intermediate to that of the grassland and forest, and tended to differ from that of the grassland (*p* = 0.092) and of the forest (*p* = 0.086). The nematode enrichment index (EI) was not significantly influenced by ecosystem type.

### Relationships between soil physico-chemical properties and soil biota

**Figure 5:**
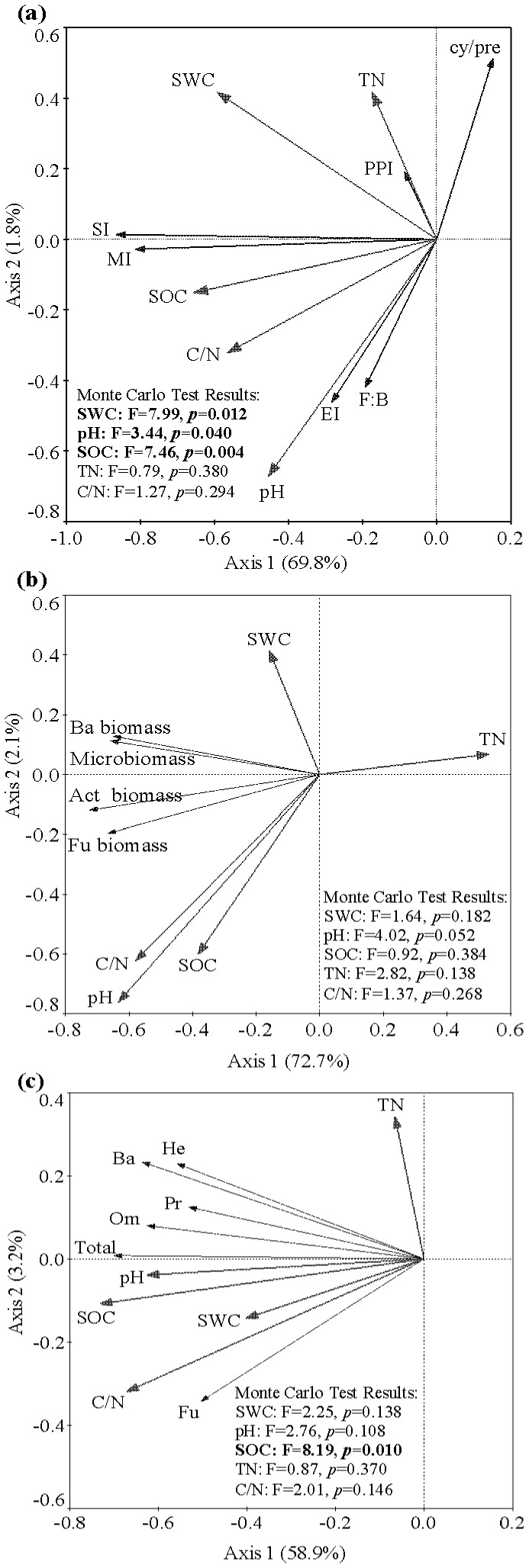
Redundancy analysis (RDA) of microbial and nematode community indices (a), microbial community biomass (b), and nematode community density (c). Ordination diagrams present species scores and environmental factor scores (vectors). Environmental factors include soil water content (SWC), soil pH, soil total nitrogen (TN), soil organic carbon (SOC), and ratio of soil organic carbon to total nitrogen (C:N). Microbial and nematode community indices include ratio of fungal biomass to bacterial biomass (F:B), cy/pre ratio of PLFAs (cy/pre), nematode maturity index (MI), plant parasite index (PPI), structure index (SI), and enrichment index (EI). Variables of microbial community biomass include total microbial biomass (Microbiomass), fungal biomass (Fu biomass), bacterial biomass (Ba biomass), and actinomycetes biomass (Act biomass). Variables of nematode community density include densities of total nematodes (Total), bacterivores (Ba), fungivores (Fu), herbivores (He), omnivores (Om), and predators (Pr).

The first two canonical axes explained about 71.6%, 51.9%, and 62.1% of the total variance of microbial and nematode community indices (Fig. 5a), microbial community biomass (Fig. 5b), and nematode community density (Fig. 5c), respectively. The microbial and nematode community indices were related to soil water content (*p* = 0.012, F = 7.99), SOC (*p* = 0.004, F = 7.46), and soil pH (*p* = 0.040, F = 3.44) (Fig. 5a). For each index, SI and MI were mainly and positively correlated with SOC; EI and F:B were mainly and positively correlated with pH; cy/pre was mainly and negatively correlated with pH; and PPI was mainly and positively correlated with TN (Fig. 5a). The microbial community biomass tended to be correlated with soil pH (*p* = 0.052, F = 4.02) (Fig. 5b). Total microbial biomass, fungal biomass, bacterial biomass, and actinomycetes biomass were mainly and negatively correlated with TN (Fig. 5b). Nematode community density was significantly related to SOC (*p* = 0.010, F = 8.19) (Fig. 5c). Total nematode density, omnivore density, and predator density were mainly and positively correlated with pH and SOC; fungivore density was mainly and positively correlated with soil water content and C/N; and herbivore density and bacterivore density were positively correlated with TN, pH, and SOC (Fig. 5c).

## Discussion

### Succession of soil microbial communities

In this study of a degraded karst region, the progressive secondary succession in vegetation was generally accompanied by a decline of the soil microbial community in that microbial biomass and other microbial variables decreased as the plant community succeeded from grassland to shrubland to forest. Consistent with our results, microbial biomass declined along a secondary succession from grassland to early stages of forest in calcareous soils in northwestern France [20]. In contrast, a previous study reported that succession from grassland to forest increased the soil microbial biomass and biodiversity in karst calcareous soils in southwest China [36]. Another study reported that bacterial phylogenetic diversity was higher but that bacterial metabolic diversity was lower in a grassland than in a forest in karst calcareous soils in southwest China [11]. Conversely, soil microbial biomass decreased with intensity of land degradation from forest to shrubland and grassland in karst calcareous soils in southwest China [37]. In these previous studies in karst areas in southwest China, the responses of soil microbial communities (mainly total microbial biomass) to vegetation succession could be ascribed to the shifts in soil organic carbon, i.e., microbial biomass was positively correlated with soil organic carbon. Although this was also the case in this study, soil organic carbon declined with plant succession. It has been well established that soil organic carbon is usually greater in grasslands than in forests [20], [38], and land use changes from grassland to forest have decreased soil carbon stocks worldwide [39]. It is also possible that the relationship between microbial communities and soil organic carbon differs among ecosystems. For instance, the relationship between the F/B ratio and soil organic carbon was positive in agricultural soils but negative in prairie soils at Fermilab, Batavia, in Illinois [40].

In the present study, the increase in the cy/pre ratio with secondary succession might indicate that the soil habitat was becoming more harsh with plant succession. Many previous studies have documented that increasing cy/pre ratios is associated with stress and starvation (e.g., anaerobic conditions, nutrient stress, and water stress) [22], [41]–[42]. Moreover, soil microbial communities in the current study were primarily influenced by soil pH, and the cy/pre ratio was negatively correlated with soil pH. Therefore, soil acidification and the deterioration of the soil habitat occurring with secondary vegetation succession might be the primary environmental factors affecting soil microbial communities. Previous studies had demonstrated that soil acidification significantly reduced the soil microbial biomasses and/or activities and soil nematode abundances [43]–[45]. In addition, many studies have reported that succession from grassland to forest decreased the soil pH [15], [46]–[48]. Soil pH in the present study was not only negatively associated with the cy/pre ratio but was also positively associated with the F:B ratio. In agreement with our results, the F:B ratio increased slightly with increasing pH as reported by [49]. However, the relationship between the F:B ratio and soil pH is inconsistent [24], [50]. Although soil total nitrogen was not influenced by vegetation type, total microbial biomass, bacterial biomass, fungal biomass, and actinomycetes biomass tended to be negatively correlated with soil TN. The reasons for this unexpected result are unclear.

### Succession of soil nematode communities

Like soil microbial biomass, soil nematode abundance showed declined patterns with respect to the progressive successional patterns of the plant communities in this study. Few studies have explored the soil nematode communities associated with plant community succession from grassland to forest or from forest to grassland. One study reported that soil nematode densities and biodiversity (i.e., plant feeders and non-plant feeders) increased with secondary succession from savanna to forest in the tropical semi-arid zone of West Africa (Senegal) [51]. Although many previous studies have compared the compositions of soil nematode communities of grasslands, shrublands, and forests, it is unclear how inferences based on ecosystem type can be applied to ecosystem succession. For example, Wasilewska (1979) reported that densities of herbivorous, omnivorous, carnivorous, and total nematodes and number of nematode genera were higher in grassland than in forest ecosystems but that densities of bacterivorous and fungivorous nematodes were similar in grassland and forest ecosystems [52]. In another study, land-use change from grassland to shrub heathland suppressed densities of nematode trophic groups (i.e., herbivores, bacterivores, fungivores, omnivores, and predators) in northwest Europe [47].

The weighted soil nematode fauna analysis showed that the nematode communities of all three ecosystems projected onto quadrat III in the nematode fauna plot, which demonstrated that the structures of the soil food webs of all three ecosystems were relatively similar [29]. However, the values of the nematode structure index (SI) decreased from grassland to shrubland to forest ecosystems, which demonstrated that the complexity of the soil food webs decreased in the order grassland > shrubland > forest. In addition, soil organic carbon was the main factor associated with total nematode density and the density of each trophic group. In agreement with our results, many studies have reported that soil organic carbon affects nematode communities [53]–[55]. Soil nematodes are also affected by spatial heterogeneity, plant community composition, and the soil environment [56]. Although the nematode plant-parasitic index (PPI) was positively correlated with soil total nitrogen, and although the enrichment index (EI) was positively correlated with pH, soil total nitrogen and EI did not significantly change with the secondary vegetation succession. Therefore, soil nematode communities might be more affected by the main soil resource (i.e., soil organic carbon) than by pH or other components of the soil habitat.

### Potential implications for land conservation and vegetation recovery in karst areas

The aim of traditional ecosystem restoration in degraded karst areas has usually been to restore forest ecosystems [4]–[5], [8], [10]. Our study clearly shows that ecosystem restoration in degraded karst areas might not necessarily be favored by secondary succession to forests. Based on the results of the current study, soil carbon storage and soil biodiversity are greater in grasslands and shrublands than in forests in degraded karst areas. The low soil organic carbon content and biota biomass associated with the forest might be explained in part by karst soils, which are generally very shallow and which can provide only low levels of nutrients and water [57]. The limitations of nutrient and water supply can be exacerbated when the live plant biomass increases during succession [58]. In addition, because, the live biomass is greater for forests than for grasslands or shrublands, natural and/or anthropogenic disruptions (e.g., disease and pest outbreak, drought, harvest, and contamination) may cause greater material loss from forests than from grasslands or shrublands. Consequently, natural and/or anthropogenic disruptions may be more damaging in forests than in grassland or shrubland ecosystems. Therefore, soil carbon and biota ought to be amended in degraded karst areas during vegetation succession or vegetation recovery.

Karst areas of southwest China, Southeast Asian, and Central America have large human populations [1]–[2]. The challenge is to provide the basic needs of these populations while protecting the fragile karst ecosystems. Agricultural development is the cause of soil erosion and rocky desertification in karst areas [1], [12]. As noted in the previous paragraph, disruptions of karst forest ecosystems might be especially destructive. Thus, the cultivation of crops and forestry does not seem to be suitable for maintaining karst ecosystems. In contrast, herbivorous animal husbandry in karst areas of southwest China has provided economic benefits to local farmers while reducing soil erosion [9]. In addition, the perennial C4 grass *Miscanthus* (i.e., the dominant genus in the grassland of the current study) has produced substantial biomass crops worldwide [59]. Another perennial C4 grass, hybrid napiergrass (*Pennisetum hydridum*), has also been widely cultivated and is mainly used for raising livestock in karst areas in southwest China and in other subtropical and tropical areas [60].

Most restoration studies have focused on soil nutrient recovery rather than on soil biota recovery, and many previous studies have used a “space-for-time substitution approach” to explore the soil nutrient status during succession from grassland to forest in karst areas. Most of these studies have reported that soil nutrients accumulate with secondary succession [11], [36]–[37] but that was not the case in the current study. This difference might be explained by the differences in the grasslands. In contrast to grasslands in the earlier studies, the studied grassland was much older and had experienced repeated wildfires.

## Conclusions

Contrary to our hypothesis, the progressive succession of vegetation occurring in karst sites in southwest China was accompanied by declines in soil biota abundances and food web complexity. The declines of the soil microbial and nematode communities might be explained by changes in soil habitat (i.e., reduced pH) and resource availability (i.e., reduced soil organic carbon). Because sustainable ecosystem management should integrate aboveground and belowground ecosystem properties and functions [61]–[62], our results indicate that soil biota amendment is necessary in karst areas during ecosystem restoration and management and establishment of grasslands in degraded karst areas are feasible. This finding provides a scientific basis for the development of herbivorous animal husbandry in karst areas that are under population pressure.
